# Modification of Male Courtship Motivation by Olfactory Habituation via the GABA_A_ Receptor in *Drosophila melanogaster*

**DOI:** 10.1371/journal.pone.0135186

**Published:** 2015-08-07

**Authors:** Shin-Ichiro Tachibana, Kazushige Touhara, Aki Ejima

**Affiliations:** 1 Career-Path Promotion Unit for Young Life Scientists, Kyoto University, Kyoto, 606–8501, Japan; 2 Department of Biology and Geosciences, Graduate School of Science, Osaka City University, Osaka, 558–8585, Japan; 3 Department of Applied Biological Chemistry, Graduate School of Agricultural and Life Sciences, The University of Tokyo, Tokyo, 113–8657, Japan; 4 ERATO Touhara Chemosensory Signal Project, JST, The University of Tokyo, Tokyo, 113–8657, Japan; AgroParisTech, FRANCE

## Abstract

A male-specific component, 11-*cis*-vaccenyl acetate (cVA) works as an anti-aphrodisiac pheromone in *Drosophila melanogaster*. The presence of cVA on a male suppresses the courtship motivation of other males and contributes to suppression of male-male homosexual courtship, while the absence of cVA on a female stimulates the sexual motivation of nearby males and enhances the male-female interaction. However, little is known how a male distinguishes the presence or absence of cVA on a target fly from either self-produced cVA or secondhand cVA from other males in the vicinity. In this study, we demonstrate that male flies have keen sensitivity to cVA; therefore, the presence of another male in the area reduces courtship toward a female. This reduced level of sexual motivation, however, could be overcome by pretest odor exposure via olfactory habituation to cVA. Real-time imaging of cVA-responsive sensory neurons using the neural activity sensor revealed that prolonged exposure to cVA decreased the levels of cVA responses in the primary olfactory center. Pharmacological and genetic screening revealed that signal transduction via GABA_A_ receptors contributed to this olfactory habituation. We also found that the habituation experience increased the copulation success of wild-type males in a group. In contrast, transgenic males, in which GABA input in a small subset of local neurons was blocked by RNAi, failed to acquire the sexual advantage conferred by habituation. Thus, we illustrate a novel phenomenon in which olfactory habituation positively affects sexual capability in a competitive environment.

## Introduction

11-*cis*-vaccenyl acetate (cVA) is a volatile male pheromone component of the fruit fly *Drosophila melanogaster*. It is a male-specific lipid, synthesized in the ejaculatory duct [1, 2], and a single mature male possesses 200 ng to 2 μg cVA on its cuticular surface [3, 4]. The presence or absence of cVA is used for sex discrimination and controls reproductive behaviors of males and females [5]. cVA is an anti-aphrodisiac for a male and inhibits courtship [4–8], while cVA emitted by a courting male is an aphrodisiac for a female and enhances copulation receptivity [8]. cVA is also known to trigger intraspecific aggregation [3] and male-male aggression [9, 10].

The anti-aphrodisiac function of cVA not only contributes to suppressing male-male homosexual courtship [8], but also helps a male discriminate a virgin female from a mated female. Previously we found that cVA is transferred to a female during copulation with other seminal fluid components and reduces sexual attractiveness of the mated female due to the slight amount of cVA that she presents [4]. Mass spectrometry analysis of the cuticular surfaces revealed that the average amount of cVA on a single mated female 24 hours after mating was 9–200 ng [4, 6, 11, 12]. Since a mated female will not accept a second courting male and avoids further copulation, it is feasible for a male to judge the partner's mating status by the presence or absence of cVA and adjust his courtship activity accordingly. However, it has been unclear how male flies distinguish presence or absence of a tiny amount of cVA on the female target fly from other nearby males in a complex olfactory environment and make an appropriate courtship decision.

In insects including *Drosophila*, odors are initially received by odorant receptors (ORs) expressed on the surface of olfactory receptor neurons (ORNs) located on the antennae. ORNs expressing any given OR project their axons to a specific glomerulus in the antennal lobe (AL), the primary olfactory center. These axons form synaptic contacts with projection neurons (PNs), which send axons to the mushroom body, and local neurons (LNs), which make connections between multiple glomeruli [13]. Thus far, two ORs, Or65a and Or67d, have been identified as cVA receptors [14]. Among these, Or67d function was shown to be required for the acute aggression response to cVA, while the chronic function of Or65a neurons was crucial for social suppression of aggression [10]. Chronic suppression of Or65a neuron activity, but not Or67d activity, also resulted in an impaired cVA response for courtship inhibition [4]. Conversely, the lack of Or67d receptor is sufficient to block the acute control of courtship behavior [8]. Recently, Or65a and Or67d receptors were found to have distinct requirements for an odor-binding protein for their activities, which could be biased by high concentrations of cVA [15], indicating functional subdivision of these two receptors.

In this study, we investigated how males respond to cVA in a variable olfactory environment and how this leads to adaptive courtship decisions. First, we found that the presence of another male or cVA in the area reduces the courtship motivation of a male toward a female. This inhibition could be overcome by pretest odor exposure and olfactory habituation to cVA or male odor. Real-time imaging of Or67d ORNs, which have high cVA sensitivity, using the neural activity marker GCaMP, revealed that prolonged exposure to cVA in the environment decreased the levels of cVA responses in the AL. Pharmacological and genetic screening indicated that GABA_A_ receptors on a small subset of LNs contributed to olfactory habituation. We also discovered that habituation allowed wild-type males to gain a sexual advantage in a competitive courtship environment that GABA_A_ mutant males failed to acquire.

## Results

### Presence of another male suppresses male courtship

**Fig 1:**
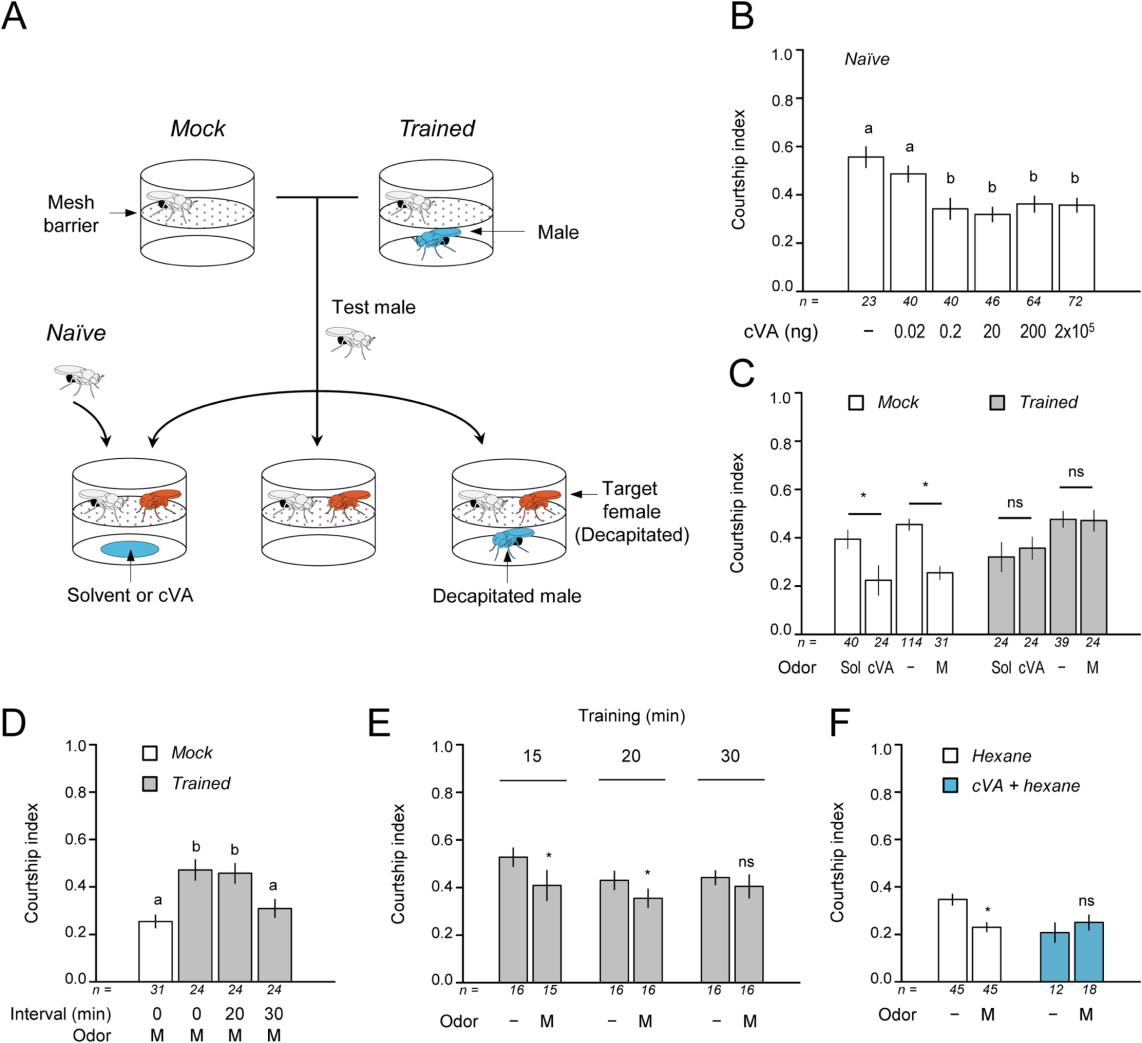
Male courtship is suppressed by the presence of cVA or another male. (A) Schematic of the assays using the double-layered chamber for measuring male courtship behavior. The upper cell of the chamber housed a test male for odor training and courtship testing and the lower cell below the mesh barrier contained cVA or a decapitated male as an odor source. For pretest odor training, a test male was housed in the upper cell without (Mock) or with (Trained) another male fly in the lower cell for 60 minutes. It was then transferred to a test chamber to be tested for the courtship level toward a decapitated virgin female as a courtship partner in the presence/absence of cVA or a decapitated male in the lower cell. As a control, an unexperienced male (Naïve) was tested. Flies and the filter paper are not to scale in the cartoon. (B) Courtship index of male flies exposed to various amount of exogenous cVA. cVA concentration of ≥0.2 ng suppressed courtship behavior of the test males. (C) Courtship index under a variety of odor sources between the mock and trained males. The courtship behavior of the untrained mock males was suppressed by the presence of 200 ng cVA compared with the presence of hexane solvent (Sol) or a decapitated male (M) compared with no male (-) in the lower cell. In contrast, the trained male showed undistinguished levels of courtship behavior regardless of the presence of the decapitated male (M) or cVA after the male-odor experience. (D) The training effect was sustained for 20 minutes after the training but not 30 minutes. The data with no interval were diverted from C as reference. (E) The odor experience for 30 minutes led to olfactory habituation, while 15 minutes or 20 minutes were insufficient to produce this effect. (F) Chronic exposure to cVA also led to odor habituation. The test males were individually reared in a test tube containing a glass capillary filled with 2 μg of cVA in 15 μl hexane or 15 μl hexane alone for 5 days. Hexane was allowed to evaporate for 2 minutes before introduction into the tube. In B-F, bars indicate the mean ± SEM. Significant differences are denoted by letters in B and D (Tukey HSD test). *, *P* < 0.05; ns, not significant in C, E, and F (Student's t-test).

To determine the anti-aphrodisiac potency of cVA, we performed a behavioral assay using a double-layered chamber (Fig 1A) in which a naïve male housed in the upper cell was exposed to various amounts of cVA in the lower cell under the mesh barrier and examined for its behavior toward a virgin female as a courtship target housed in the upper cell. We found that virgin females with no or as little as 0.02 ng cVA elicited high levels of courtship behavior from the males, while the presence of cVA over the mesh barrier decreased the courtship level of the males (Fig 1B). In the previous studies, 150 ng was demonstrated to be the minimum cVA necessary for courtship suppression [4, 7, 8, 12, 16–19]. In our assay system, 0.2 ng cVA was enough to cause a significant suppression of courtship behavior (Fig 1B), demonstrating the keen sensitivity of males to cVA. The power of suppression was consistent, as shown by no significant differences between courtship levels in the presence of various amounts of cVA except 0.02 ng (*P* > 0.05, Fig 1B). We also confirmed that the presence of not only synthetic cVA but also another male as an odor source in the lower cell of the chamber reduced courtship behavior of the test male (Fig 1C mock).

### Pretest pheromone experience changes behavioral response

This keen sensitivity to cVA raises the question about how a male fly distinguishes a tiny amount of exogenous cVA on a mated female from its self-produced cVA and how a male maintains high courtship in the presence of male competitors. One potential mechanism is habituation, defined as a decrement of behavioral response to a stimulus after repeated exposures, that allows an animal to filter out constant stimuli and focus on novel and/or variant stimuli.

To determine if the cVA responses in male courtship habituated, we preexposed males to the odor of another male for 60 minutes and tested their subsequent courtship responses to cVA or male odor (Fig 1A). As opposed to the control males that were left alone in a chamber and showed courtship suppression in the presence of either male odor or cVA (Fig 1C mock), the trained males that had been exposed to male odor retained high courtship activity regardless of the presence of a male or cVA (Fig 1C trained). The courtship latencies, defined as the time delay between pairing and first courtship performance of the male, were significantly delayed in control males in the presence of another male or cVA while the trained male started courtship with no delay (S1 Fig). Since the experiments were performed under dim red lights, a condition in which the flies cannot use visual cues, this implies that the males relied on olfaction to notice the presence of the target female and initiate courtship. These results suggest that olfactory processing was affected by the male-odor habituation experience.

The behavioral habituation to male odor remained at least 20 minutes after the odor training, but by 30 minutes males had recovered from the pre-exposure experience and again showed reduced levels of courtship to females paired with male odor, equivalent to that of the control mock males (Fig 1D). We confirmed that 30 minutes of training with either male odor or 200 ng of cVA was enough to change the behavioral responses to the concurrent presence of male odor or cVA (Fig 1E and S2 Fig). We also found that chronic cVA exposure for 5 to 6 days achieved the same effect (Fig 1F). However, 15 or 20 minutes of exposure was not enough to effectively modify the inhibition in our system.

### Intensity of cVA odor stimulus determines Or67d ORNs response levels

To determine how cVA-sensitive ORNs respond to various amounts of cVA and how ORN responses are dynamically controlled, we first performed real-time imaging using the Ca^+^-sensitive fluorescent marker (GCaMP3.0) expressed in cVA-responsive Or67d ORNs [8, 20] to examine dose-dependent neural activity upon cVA stimulation. The neural responses of ORNs and PNs to cVA have been monitored by either electrophysiology or *in vivo* imaging using relatively high concentrations of cVA: 0.1–10% [21], 1% [22], 1–100% [23], 0.1–100% [24], or 1–100% [8]. In these experiments, the final odor concentration after travelling through the delivery tubes was unclear.

**Fig 2:**
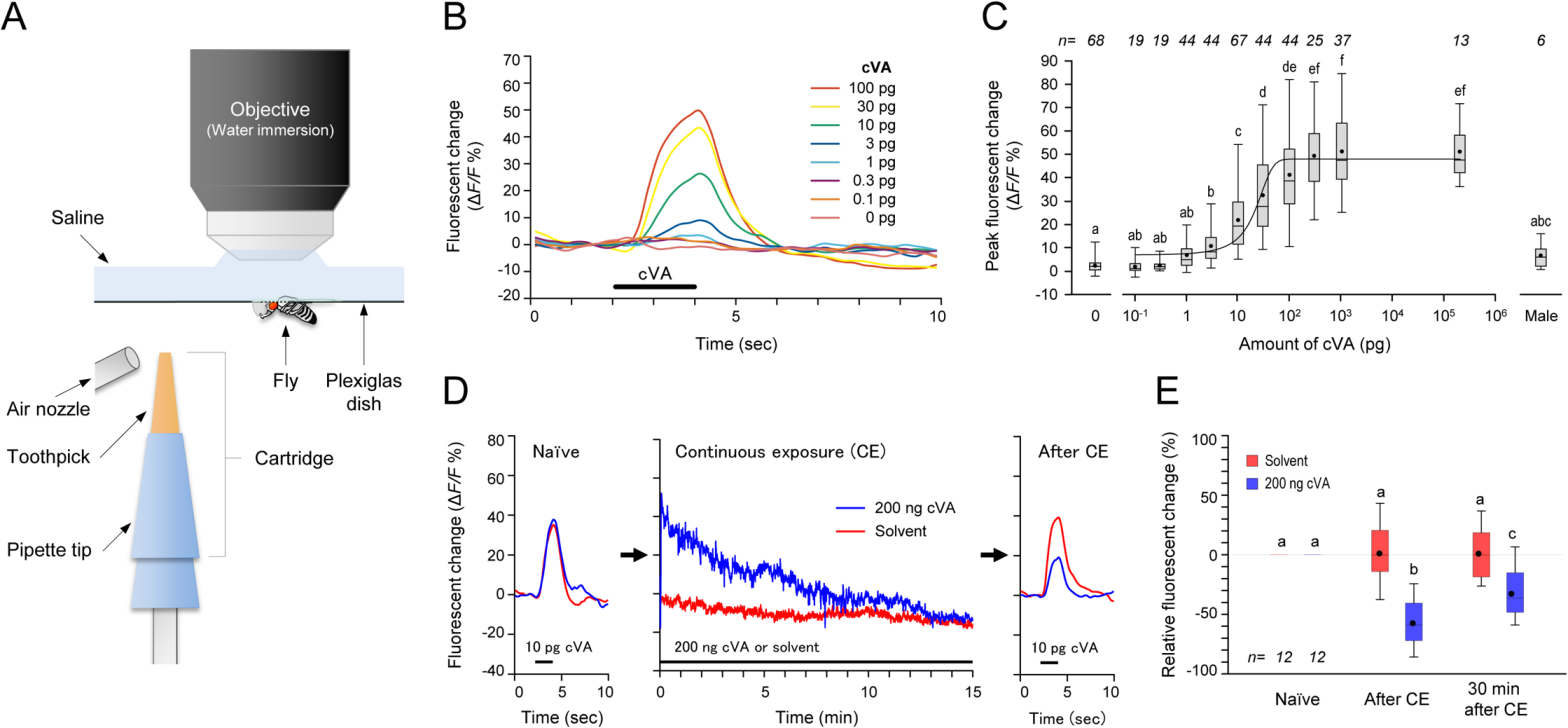
Dose-dependent cVA response and desensitization by prolonged stimulation in Or67d ORNs. (A) Odor stimulation system with *in vivo* imaging of neural responses in the DA1 glomeruli (see Methods and S3 Fig). Neural responses to cVA were monitored with a fluorescent microscope upon odor stimulation. cVA was applied on the toothpick of the cartridge and an air puff from the air nozzle delivered the volatilized cVA to the antennae of the fly. (B) Representative fluorescent changes evoked by odor stimulation of various amounts of cVA (0 to 100 pg in this case) in a male fly. (C) Peak fluorescent changes in response to cVA and a live male. Peak values of the transient fluorescent change in response to odor stimulation are shown as box plots representing 25th, 50th, and 75th percentiles with whiskers from minimum to maximum and circles (mean). A logistic curve was plotted based on mean values of the peak fluorescent changes in each amount of cVA (see Methods). EC_50_ is 27.5% in 22.3 pg cVA. Odor stimulation using a live male is shown in S4 Fig. (D) Representative neural responses to 10 pg cVA of the Or67d ORNs before (Naïve) and after continuous exposure (After CE) to 200 ng cVA (blue) or hexane solvent control (red) for 15 minutes. During the 15 minutes CE, the response to 200 ng cVA was decreased and thereafter the acute response to 10 pg cVA also decreased. (E) Peak responses to 10 pg cVA before (Naïve), immediately after (After CE), and 30 minutes after the continuous exposure (30 minutes after CE).

In this study, we developed a novel real-time imaging set-up to monitor calcium responses to a wide range of odor amounts presented in front of the object male (Fig 2A and S3A–S3C Fig). The stimulant odor was applied on the tip of toothpick positioned 25 mm in front of the subject male, and a gentle air puff was delivered for 2 seconds from an air nozzle tip 5 mm from the toothpick tip. Responses were directly monitored in the DA1 glomerulus of the brain where the cVA-responsive Or67d ORNs project their axons [8, 25, 26] (S3D and S3E Fig). The intensity of GCaMP fluorescence increased after cVA delivery via the air puff and returned to the basal level after turning off the air puff with less than a 2-second delay (Fig 2B). Application of 10 pg cVA was enough to lead to a significant increase in peak fluorescence compared with the solvent-only 0 pg control (Fig 2C). The level of neural response increased as the amount of applied cVA increased and reached a plateau at 100 pg cVA (Fig 2C). This indicates that odor strength is transmitted as the intensity of the neural responses of the ORNs in the AL. As a reference, we also monitored the response to the odor derived from a living wing-amputated male fixed on the toothpick (S4 Fig). The mean fluorescence change upon male odor stimulation was 6.6%, which is comparable to the intensity of 0 to 10 pg cVA (Fig 2C).

### Continuous cVA exposure causes desensitization of the Or67d ORN response

We then examined how the neural responses of Or67d ORNs were affected by continuous exposure to cVA using real-time imaging. After a brief 10 pg cVA stimulation, the subject fly received 200 ng cVA continuously for 15 minutes and was tested for response to 10 pg cVA. During continuous exposure (CE, Fig 2D), fluorescent intensity in the DA1 glomerulus gradually decreased over time and finally reached the basal level, which was matched by solvent-only exposure at 15 minutes. After the continuous cVA exposure, the intensity of acute response to 10 pg cVA declined by about 58% compared with that observed before CE and about 59% compared with that observed with the solvent control (Fig 2E), clearly showing the desensitization phenomenon. This reduced response started to recover 30 minutes after the cVA exposure terminated with about 25% increase in the intensity (Fig 2E).

### GABA input is required for neural desensitization of Or67d ORNs

**Fig 3:**
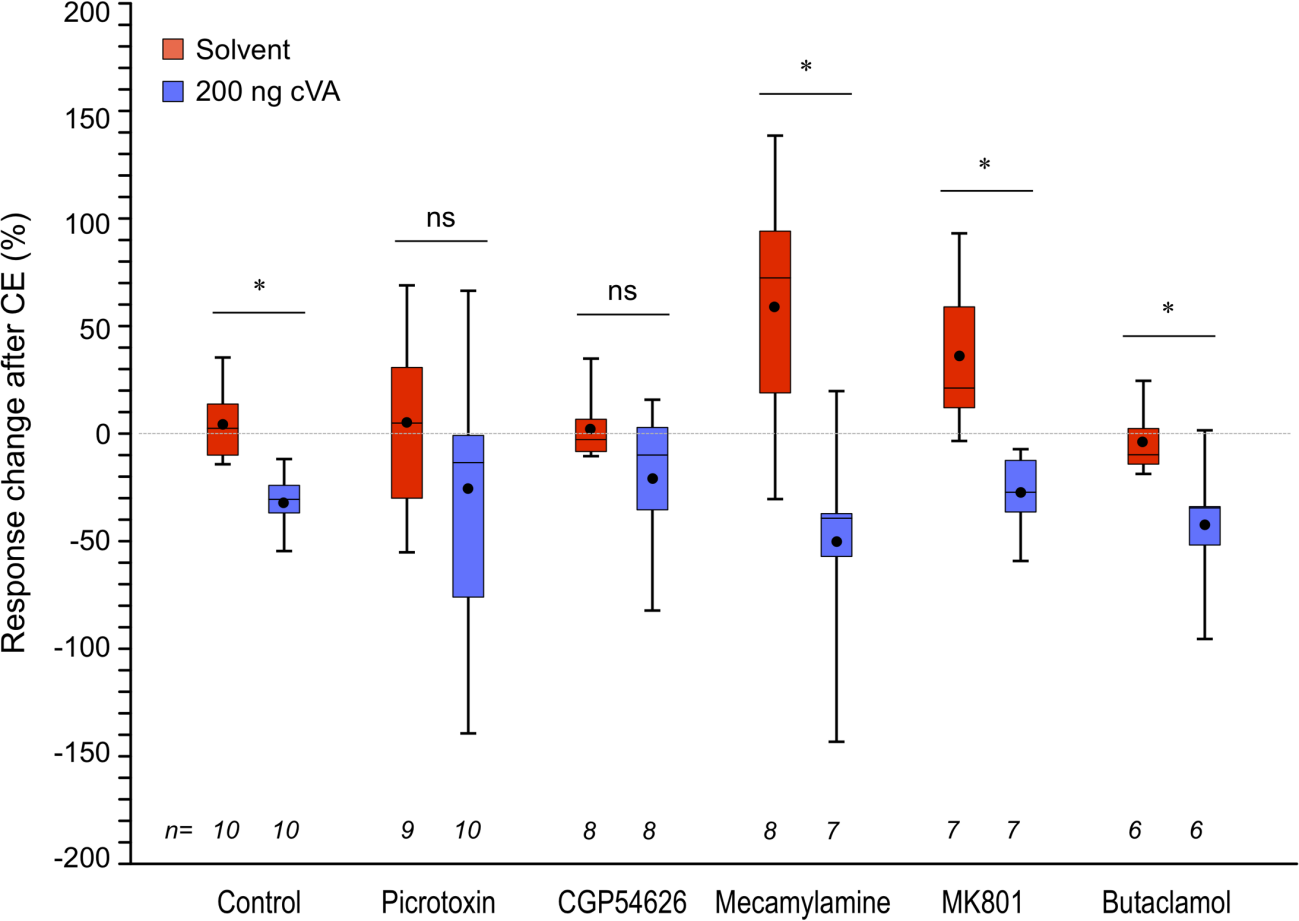
GABA input is required for neural desensitization of Or67d ORNs. Effects of five neurotransmitter antagonists on the neural desensitization driven by the odor exposure were examined. The response changes after each CE were calculated by subtraction of values of relative fluorescent change of "Dosed before CE" from that "after CE" in individual flies (S5B–S5G Fig). Picrotoxin, CGP54626, Mecamylamine, MK801, and Butaclamol were used as antagonists of GABA_A_, GABA_B_, Nicotinic acetylcholine, NMDA, and dopamine receptors, respectively. Box plots represent 25th, 50th, and 75th percentiles with whiskers from minimum to maximum and mean with circles. Significant differences are denoted by *, *P* < 0.05; ns, not significant (Student's t-test). Experimental procedure including the drug application is referred in S5 Fig.

We next performed pharmacological screening to identify the neurotransmitter(s) involved in the neural desensitization driven by cVA exposure. As a control for the drug application into the saline bath surrounding the exposed fly brain (S5A Fig), we exchanged saline alone and found that bath exchange itself decreased the responses to 10 pg cVA (S5B Fig, Dosed before CE). After the continuous cVA exposure, the response was further decreased while there was no change detected after the solvent exposure (S5B Fig, After CE). The amplitude of the CE effect, apart from the general effect of dosing treatment, was calculated by subtraction of the value of relative fluorescent change "before CE" from that "after CE" (S5 Fig) in each individual and summarized in Fig 3. In the fly that received bath exchange with no drug, the "CE effect" value after cVA CE (blue) was significantly smaller than that after the solvent exposure (red), demonstrating that the cVA CE led to the neural desensitization also in this control fly.

Within the AL, LNs are mainly GABAergic and glutamatergic [27–31], a few are cholinergic [32, 33], and a few more are dopaminergic [30]. Conversely, ORNs and PNs are cholinergic [34, 35] and the presence and/or function of several other neurotransmitters and neuropeptides, e.g., serotonin, tachykinin, and small neuropeptide F (sNPF), has also been indicated [36–38]. In our activity analysis, the application of five neurotransmitter antagonists showed significant effects in the response to cVA after CE (Fig 3, *P* < 0.0001; d.f. = 1 in CE condition, *P* < 0.05; d.f. = 5 in interaction with the application of drugs and CE condition by two-way ANOVA). On closer inspection in the effect of each drug, the application of GABA antagonists, picrotoxin against the GABA_A_ receptor, and CGP54626 against the GABA_B_ receptor resulted in the unaffected cVA responses after cVA exposure, indicating the impaired action of the neural desensitization. It should be noted, however, that the response changes after each CE in the presence of these drugs remained at the same levels as those in the control (*P* > 0.05 by Student’s t-test with Holm's correction within each CE condition), implying the alternative possibility that the response variations driven by the application of these drugs (S5C and S5D Fig) indirectly diminished the cVA CE effect. Therefore, we performed the further analysis using genetic tools to confirm the function of GABA in the neural desensitization in the following section. On the other hand, though the involvement of glutamate and dopamine for information processing within the AL has been indicated [30, 31], antagonists against *N-*methyl-d-aspartate (NMDA) (MK 801), and dopamine (butaclamol) receptors did not affect the neural desensitization. Meanwhile, the inhibition of acetylcholine inputs by mecamylamine had the general stimulatory effect, which gradually increased the basal response levels of the neurons (S5E Fig) and consequently increased the "CE effect" value in the solvent-exposed control fly (Fig 3, *P* < 0.05 compared to that of no-drug control by Student’s t-test with Holm's correction), while the desensitization was not affected (*P* < 0.05 by Student’s t-test with Holm's correction, comparison between two CE conditions). Altogether, it was suggested that the cVA CE desensitized the olfactory neurons independently of actions of glutamate, dopamine or acetylcholine.

### cVA olfactory habituation requires GABA inputs into LNs

**Fig 4:**
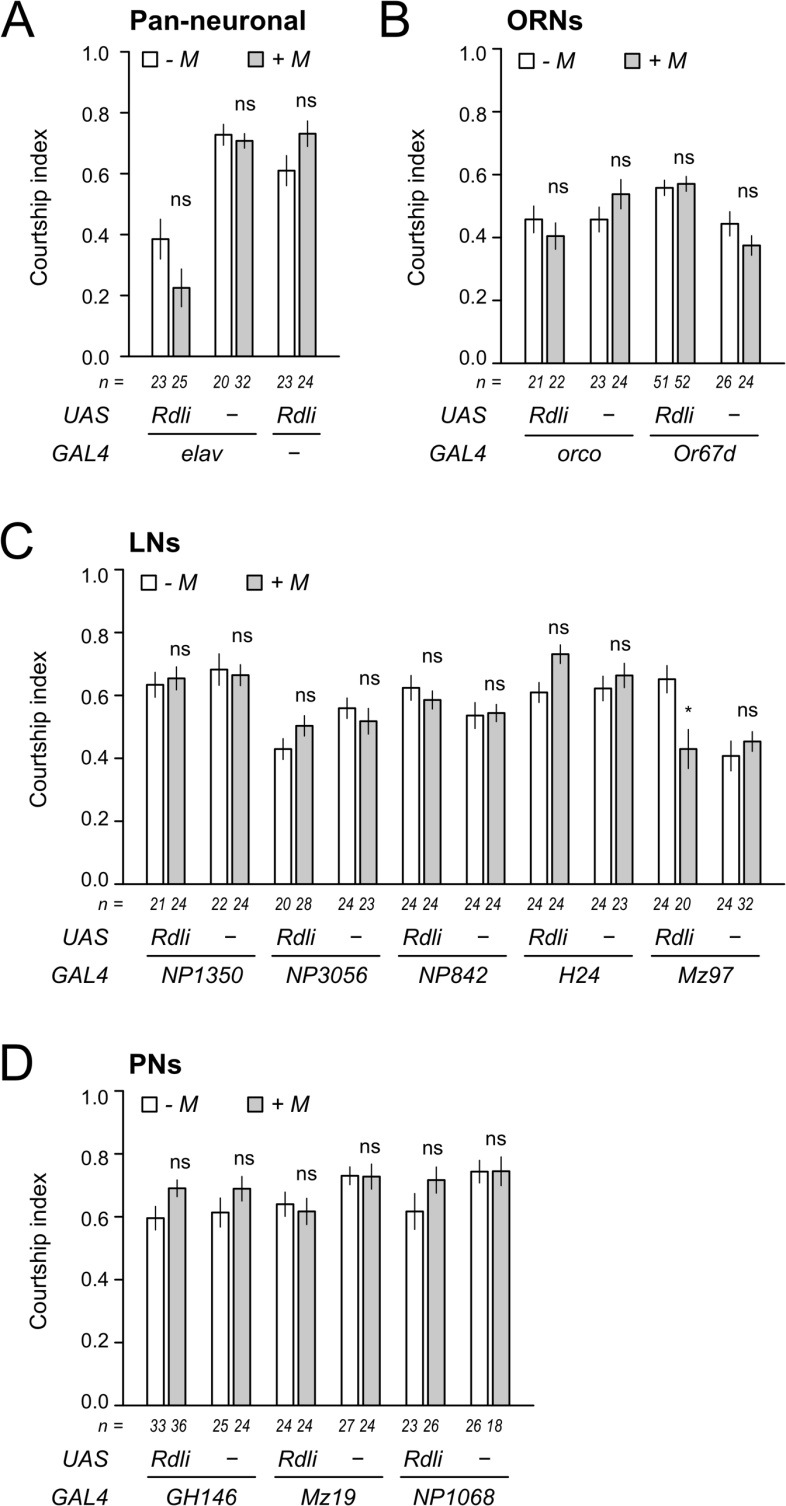
The GABA_A_ receptor in LNs is involved in the behavioral habituation to cVA. The defect of behavioral habituation to cVA was analyzed by interference of the expression of GABA_**A**_ receptors in a major class of olfactory neurons using pan-neuronal (A), ORNs (B), LNs (C), and PNs (D) drivers. The male flies were all trained with the male odor for 60 minutes, and then courtship responses to the females without (- M) or with (+ M) male odor in the lower cell of the chamber were measured (see Fig 1A). Bars indicate mean ±SEM. *, *P* < 0.05; ns, not significant from the Student's t-test with Holm's correction.

To confirm the involvement of GABA in the plastic control of odor sensitivity and also determine the olfactory neurons that received GABA input for the action, we performed genetic screening using RNAi. Expressing RNAi against the GABA_A_ receptor *Rdl* under the control of various GAL4 drivers, we searched for conditions in which male odor sensitivity was uninfluenced by habituation. Using a pan-neural driver (Fig 4A), two ORN drivers (Fig 4B), five LN drivers (Fig 4C), and three PN drivers (Fig 4D), the expression of *Rdl* was suppressed by *Rdl*-RNAi and its effect on the courtship responses after habituation training was examined. Then we found that the *Rdl* suppression by the LN driver Mz97 led to impaired habituation as shown by the suppressed courtship in the presence of male odor even after male odor training (*P* < 0.05 Student's t-test with Holm's correction, comparison between two test conditions; Fig 4C). The Mz97 strain marks 3–4 AL-associated neurons whose cell types are not identified yet [39]. This nullification of habituation training was not found in males expressing the RNAi constructs with any other drivers (Fig 4), indicating that GABA inputs acting through GABA_A_ receptor on a specific subset of LNs marked by Mz97 play an important role in olfactory habituation to cVA.

### Olfactory habituation provides a sexual advantage in competitive courtship situations

**Fig 5:**
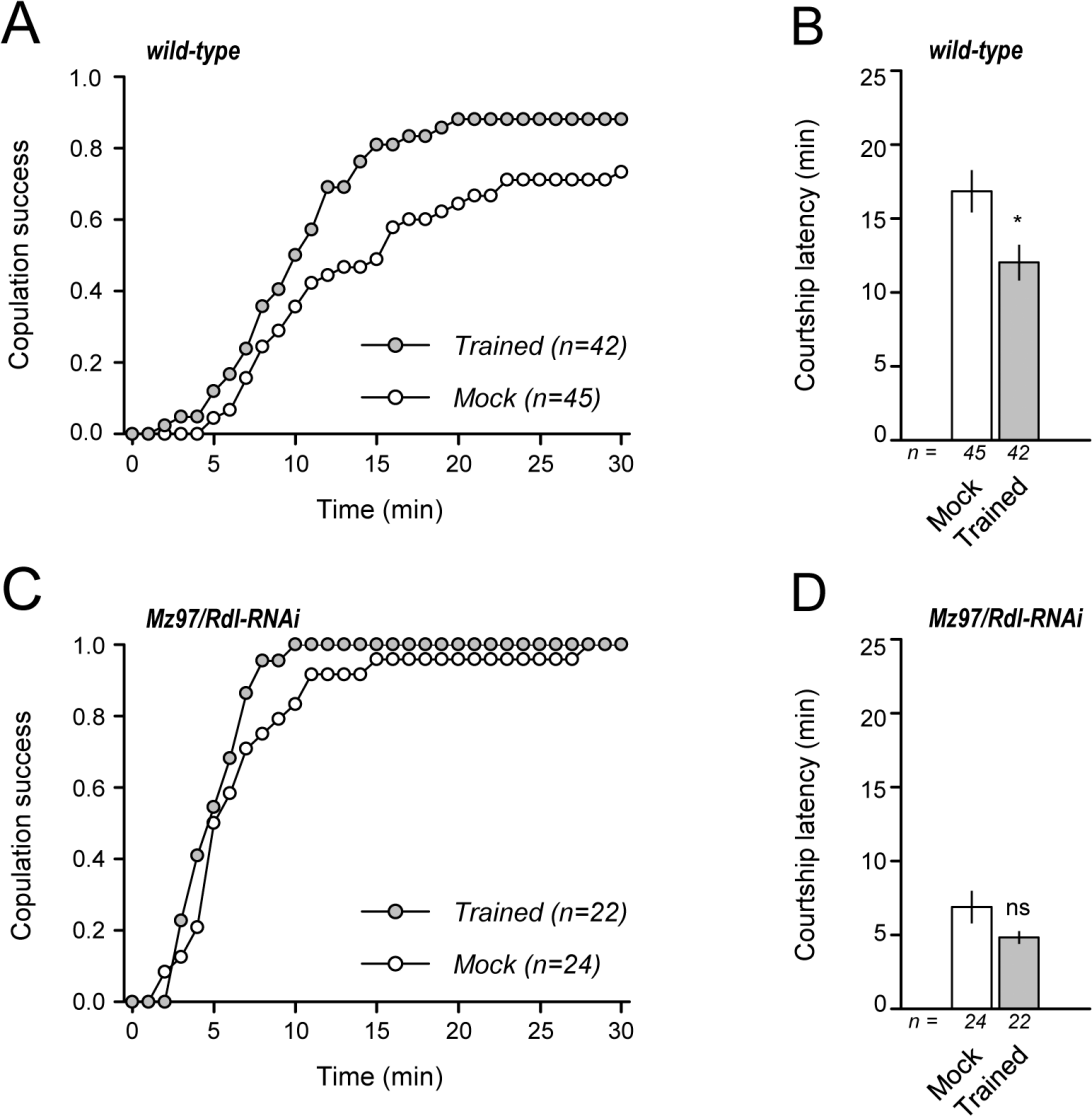
Odor habituation provides sexual advantage. The numbers of successful copulations, copulation latency, and time lag between pairing and copulation, were measured in the males of wild-type (A, B) and the GABA_A_ receptor mutant, Mz97/*Rdl*-RNAi (C, D). During the 30-minute observation, the trained wild-type males showed a high ratio of copulation success (A) and decreased latency (B) compared with that of the control mock males. Conversely, the GABA_A_ receptor mutant males, in which GABA input into a subset of LNs was blocked by *Rdl*-RNAi, showed undistinguished levels of copulation success and latency in the competitive courtship condition (C, D). *, *P* < 0.05; ns, not significant from the Student's t-test.

Lastly, to determine if there was a reproductive benefit of male-odor habituation, we measured copulation success of males with or without habituation training. In a competitive situation, in which a test male was paired with a female and another male, the trained wild-type males showed greater copulation success compared with that of control males (Fig 5A). Copulation latency, which is the delay in time until copulation initiation, was significantly smaller for the trained male (Fig 5B), suggesting the pretest experience provided the males some sexual advantages in the competitive courtship situation in which the male meets a potential mating partner in the presence of a sexual competitor. In contrast, the transgenic males in which GABA input into the LNs was blocked by *Rdl*-RNAi showed no difference in copulation success and courtship latency regardless of the previous odor experience (Fig 5C and 5D), indicating no sexual benefit acquired in these mutant males. Thus, the habituation function is crucial for their reproductive success.

## Discussion

### Biological benefit of olfactory habituation

Habituation is defined as decrement of behavioral response to an iterative stimulus and is considered as the simplest form of learning and memory; it allows animals to filter out irrelevant stimuli of diminishing relevance and focus selectively on important stimuli [40–44].

In *Drosophila melanogaster*, habituation has been studied in genetic, molecular, and neural controls of escape-related behaviors, e.g., jump, startle, leg movement, or escape upon strong odor stimulation [28, 45–47]. However, the biological relevance of habituation for the animal in terms of survival and reproductive probability has received less attention. In this study, we demonstrated that the olfactory habituation to male-odor or cVA, a courtship inhibitory pheromone, allows a male to maintain a high level of sexual motivation and therefore increase the probability of copulation success in a competitive environment (Fig 5). This is the first report of habituation positively contributing to sexual advantage in a semi-natural experimental condition.

We suspect that cVA habituation provides benefit to the male in the wild since the amount of cVA present in the environment is variable. A newly emerged immature male contains no detectable cVA, but 4 hours later, small amounts of cVA (20 ng) appear and gradually increase to reach as much as 2.9 μg at 4 weeks of age [3]. The amount of cVA on the mated female varies depending on the interval after copulation [6]. There are also strain-dependent differences found in wild-caught males [48]. Under such variable odor conditions including its own smell, plasticity in cVA sensitivity could provide a reasonable benefit to the animal in making adaptive behavioral choices.

Habituation to cVA influenced male behavior only in the context of female courtship—it did not increase male-male homosexual courtship behavior (S6 Fig). Although the presence of cVA has been considered to contribute to sex discrimination, it is not the only anti-aphrodisiac factor, and other "maleness" factors, including behavioral pattern [49] or gustatory information [50, 51], could compensate for the reduced levels of odor sensitivity caused by habituation.

### Molecular control of olfactory habituation

From genetic studies, the involvement of several intracellular- and intercellular-signaling molecules has been indicated. Mutations of *rutabaga* gene that diminish cAMP synthesis reduced habituation in escape response upon visual stimulus, while *dunce* mutations that increase cAMP levels enhanced the habituation [45]. In odor avoidance responses, ryanodine receptor and inositol (1,4,5)-trisphosphate receptor in ORN [52]; *rutabaga*, *synapsin*, vesicular glutamate transporter, and calcium/calmodulin-dependent protein kinase II (CaMKII) in LNs [47, 53]; and Ataxin-2, GABA, and glutamate inputs in PNs [46, 53] were required for habituation. Conversely, only a few reports demonstrate habituation in male courtship behavior in which exposure to the odor of young males led to reduced behavioral responses toward young males [54–56]. The involvement of *dunce*, *inactive*, *amnesiac*, or *rutabaga* has been suggested for this courtship control; however, their loci of action in the brain were unclear due to the non-conditional genetic techniques at the time [54, 55]. In this study, we demonstrated that continuous odor exposure decreased the response intensity of the Or67d ORNs in the axon terminals and identified the involvement of the inhibitory neurotransmitter GABA by pharmacological screening. In our real-time imaging, application of both GABA blockers, picrotoxin (GABA_A_ receptor antagonist) and CGP54626 (GABA_B_ receptor antagonist), [27, 57] disturbed the neural desensitization (Fig 3). On the other hand, application of mecamylamine, a nicotinic acetylcholine receptor antagonist [57], MK801, a NMDA receptor antagonist [58], and butaclamol, a dopamine receptor antagonist [59], had no impact on odor desensitization (Fig 3), while mecamylamine increased basal levels of neural activities apart from the desensitization (S5E Fig). This is consistent with previous studies showing the actions of the neurotransmitters nicotinic acetylcholine and GABA in the AL in odor avoidance behaviors [28, 53].

### Neural network for olfactory habituation

Olfactory information is transmitted from ORNs to PNs, potentially with modification from LNs in the AL glomeruli, and then conveyed to higher brain regions, the lateral horn and mushroom body [60–62]. Previously, electroantennogram recordings revealed that odor preexposure weakened the response amplitudes of ORNs in the antenna, indicating local action of intracellular modification in the sensory sensillum [63, 64]. Meanwhile, other reports suggested that no modification of olfactory transduction necessarily accompanied the habituation in odor avoidance responses [65, 66]. It was also reported that the habituation in aggressive behavior via cVA was not accompanied by modification of dendritic receptor activity in the antenna, indicating contribution of higher brain function for the context dependent behavioral changes [10]. Recent evidence indicates that intercellular processing of odor information in the AL has a critical role in the olfactory habituation of escape responses [28, 47, 53], which could be applied to the cVA habituation in courtship control also.

Using real-time imaging, we found that desensitization of the Or67d ORNs was sensitive to neurotransmitter antagonists. This suggests that the site of action of habituation is within the neural network in the AL. To determine which olfactory neurons required GABA input for olfactory habituation, we expressed RNAi constructs against the *Rdl* GABA_A_ receptor using various GAL4 drivers and found that habituation was blocked when expression of the GABA_A_ receptor *Rdl* was disrupted under the control of Mz97 driver (Fig 4). The Mz97 strain expresses GAL4 in approximately three neurons situated in the area ventrolateral to the AL and in a single neuron that arborizes in multiple AL glomeruli including DA1, and terminates in the antennal mechanosensory and motor center (AMMC) of the contralateral hemisphere [39]. Therefore, it is feasible to consider that this Mz97-LN innervating DA1 glomerulus was involved in the desensitization of Or67d ORNs.

Previous reports showed that GABA input into PNs from LNs was critical for habituation of the avoidance response to harmful materials such as carbon dioxide and ethyl butyrate, indicating that the habituation was the amplified activity of inhibitory LNs (iLNs) [28, 53]. In contrast, in our RNAi screening, expression of the RNAi constructs against the GABA_A_ receptor in PNs under the same GH146 GAL4 driver showed no effect on the male-odor habituation (Fig 4D), implying the distinct processing for habituation of escape behavior and courtship control. Meanwhile, in our real-time imaging, continuous cVA stimulus led to suppression of response amplitudes of the Or67d ORNs (Fig 2D and 2E), which was sensitive to several neurotransmitter antagonists, suggesting the action of inhibitory feedback to the ORNs within the AL. However, we found no impairment of the male-odor habituation in the transgenic males, in which GABA input into the Or67d ORNs was blocked. This indicates that other inhibitory machinery mediates the desensitization of the Or67d ORNs.

**Fig 6:**
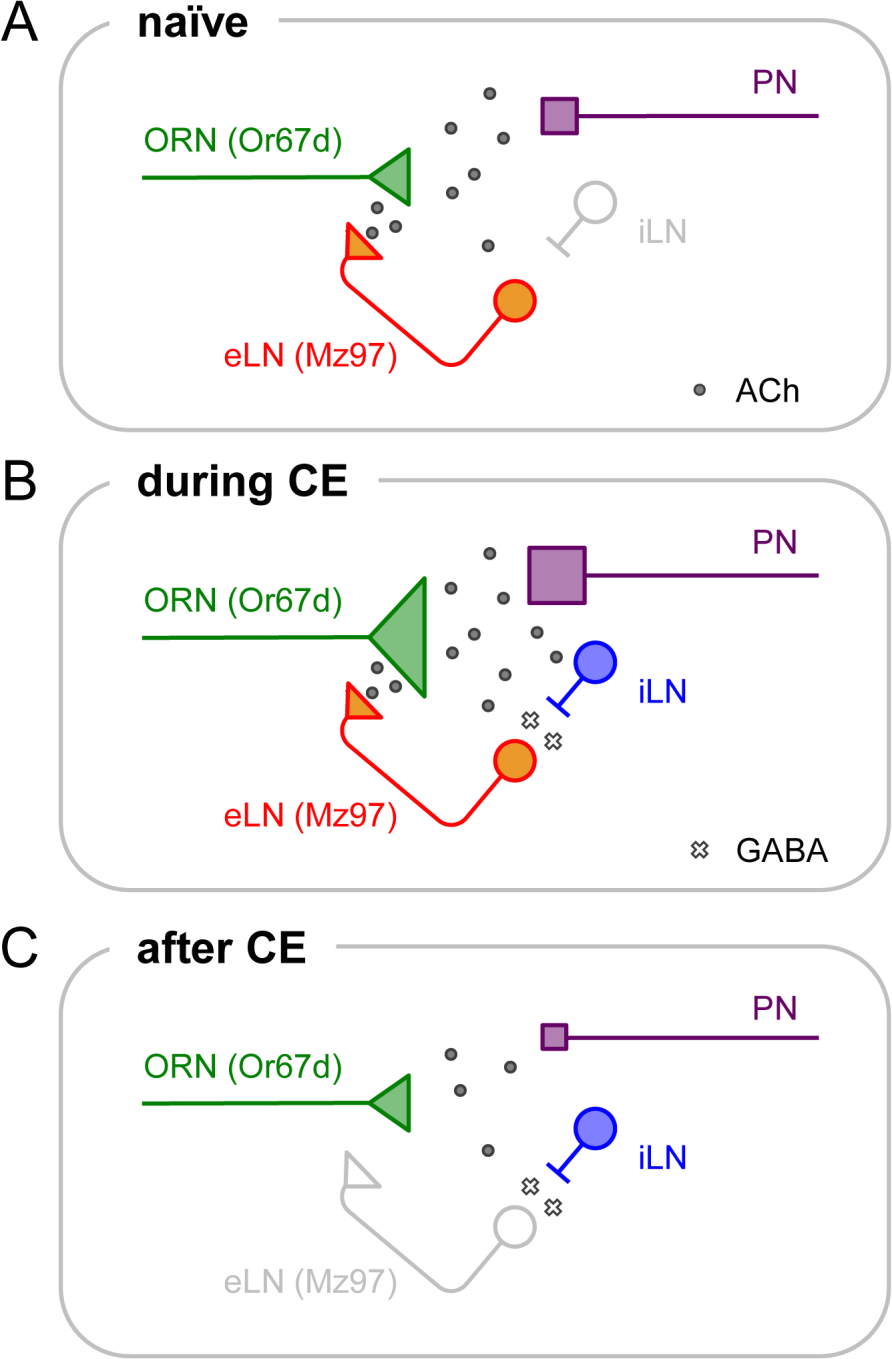
Recurrent inhibitory circuit plastically reduces signal intensity. (A) The Mz97 LNs provide presynaptic excitatory inputs into the Or67d ORNs in a naïve fly. (B) Continuous odor exposure facilitates the iLNs that provide inhibitory inputs to the Mz97 LNs. (C) Suppression of the Mz97 activity weakens the odor signal from the ORNs and downstream behavioral responses.

One of the possible mechanisms for the male-odor habituation is recurrent inhibition, defined as activity dependent regulation of excitatory neurons by inhibitory interneuron as shown in the siphon withdrawal reflex of *Aplysia* [67–71]. In the AL of *Drosophila*, most of the LNs are picrotoxin sensitive, and inhibition between LNs has been predicted [27, 57, 72]. The fact that interference of the GABA input into the Mz97 LNs retained the male-odor sensitivity even after habituation training (Fig 4) allows us to predict that the Mz97 LNs are excitatory (eLNs) in a naïve fly, providing positive feedback to the presynaptic terminals of the ORNs to facilitate the odor signaling when the ORNs are activated (Fig 6A). Considering eLNs in the AL show spontaneous activity [57], it is possible that the eLNs also chronically stimulate cVA signaling and maintain low courtship motivation independent of cVA exposure. The involvement of the eLNs is also supported by our real-time imaging results; the desensitization of the Or67d ORNs was disturbed by the application of picrotoxin and CGP54626 (Fig 3) as were the activities of the eLNs in a previous study [57]. In summation, we propose a recurrent circuit model as illustrated in Fig 6. First, the Mz97 LNs provide presynaptic excitatory inputs into the Or67d ORNs in a naïve fly (Fig 6A). Upon continuous odor stimulation, the enhanced odor signal facilitates the GABAergic iLNs that have inhibitory connections to the Mz97 LNs (Fig 6B). Subsequently, suppression of the Mz97 activity (presynaptic excitation) weakens the odor signal from the ORNs and eventually decreases downstream behavioral responses (Fig 6C).

In addition to GABA, the involvement of acetylcholine for information processing was indicated by the pharmacological screening (S5E Fig). Considering that the vast majority of the AL neurons, ORNs, some LNs and PNs, are cholinergic [33–35], it is feasible that this neurotransmitter plays a critical role to control activities of the olfactory neurons. However, in our imaging preparation, since the drug penetrated throughout the brain, we could not specify the target neurons affected by mecamylamine, a nicotinic acetylcholine receptor antagonist at this moment. Further investigation will reveal the direct molecular processing to control the activity levels of the olfactory neurons and lead to gain in reproductive success.

## Materials and Methods

### *Drosophila* stocks

Canton S (CS) flies were used as wild-type controls and courtship objects. The transgenic lines used were: *UAS-Rdli-8-10J* [73], *orco-GAL4* (previously known as *Or83b-GAL4* [74]), *NP842-GAL4*, *NP1068-GAL4*, *NP1350*, *Mz19-GAL4* [39], *NP3056-GAL4* [30], *H24-GAL4* [75], *GH146-GAL4* [32], and *Mz9-GAL47* [76]. The *Or67d-GAL4 57*.*3* strain was generously provided by Leslie Vosshall (Rockefeller University, New York, NY, USA). The RNAi stock for the GABA_A_ receptor (resistance to dieldrin, *Rdl*) gene, *UAS-Rdli-8-10J*, was obtained from Leslie Griffith (Brandeis University, Waltham, MA, USA) [77]. *NP842-GAL4* and *Mz97-GAL4* flies were generously provided by Kei Ito (The University of Tokyo, Tokyo, Japan). *GH298-GAL4* and *Mz19-GAL4* flies were generously provided by Rachel Wilson (Harvard Medical School, Boston, MA, USA). *elav* ^*C155*^ *-GAL4* (stock # 105921), *NP1350-GAL4* (stock # 112640), *NP3056-GAL4* (stock # 113080), and *NP1068-GAL4* (stock # 112482) flies were obtained from *Drosophila* Genetic Resource Center (Kyoto, Japan). *H24-GAL4* (stock # 51632), *GH146-GAL4* (stock # 30026), and *UAS-GCaMP3*.*0* (stock # 32116) flies were obtained from Bloomington *Drosophila* Stock Center (Bloomington, IN, USA).

All flies were reared on cornmeal-sucrose-agar standard medium at 25°C under a 12/12 hour light/dark cycle. Adult male virgin flies were collected within 4 hours of eclosion using CO_2_ anesthesia, and aged for 4–6 days in individual test tubes containing grape juice (Welch's grape 100, Calpis) with 10% agar until the test. Only males with intact wings were used in the experiment.

### Behavioral assays

All behavior experiments were performed under dim red lights (>700 nm), unless otherwise noted, in an environmental room (25°C, 70% humidity). A double-layered courtship chamber is a modified wheel chamber [78, 79], composed of two Plexiglas layers with 8 observation cells (8 mm in diameter, 3 mm in depth each) separated by a fine nylon mesh (Tetko, 3-180/43), which prevents the flies in the upper cell from direct contact with odor source in the lower cell (Fig 1A), and bookended by a top-layer and a bottom-layer. A 4–6-day-old male was placed with a decapitated mature (4–6-day-old) female tester as a "courtship object" in an upper cell of a double-layered chamber and its courtship performance was videotaped with a CCD camera (Allied, Guppy) for 5 minutes. The courtship index was calculated as the proportion of time a male displayed courtship action during the 5-minute observation period. Courtship latency was the time lag to the first courtship display (courtship orientation) after pairing. A latency value of 300 seconds was recorded when no courtship was performed during the 5-minute observation. cVA (Pherobank) was diluted with hexane solvent. Just before testing, 6 μl cVA solution was applied on a piece of filter paper in the lower cell of the chamber and hexane was evaporated for 2 minutes at room temperature.

For habituation training, a male was placed in the upper cell of the double-layered chamber, which was exposed for 60 minutes to the odor source placed in the lower cell. Immediately after training, males were transferred into a clean chamber and paired with a decapitated mature female tester for 5 minutes. Mock trained males were kept alone in the chamber for the first hour, and then paired with a tester.

For copulation experiments, a male was paired with an intact female and a decapitated 4–6-day-old male in a single layered chamber (8 mm in diameter, 6 mm in depth) under dim red lights. Timings of copulation and time lags until the successful copulation were recorded for 30 minutes. A latency value of 1800 seconds was given when no copulation was performed during the observation.

### *In vivo* real-time imaging of neural activity

One–five-day-old male flies that were reared in the individual test tubes mentioned above were used. The flies were anesthetized by transferring to a glass vial on ice and then restrained on a Plexiglas dish (S3A Fig) at room temperature. We fixed the wings and legs to the Plexiglas dish; the proboscis was fixed to a silver fiber with wax (S3B and S3C Fig). A piece of aluminum foil was inserted between the upper part of the fly head including antennae and the lower part of the fly body and fixed by epoxy sealing (S3B and S3C Fig). The fly fixed on the Plexiglas dish was left in a plastic culture dish with moistened filter papers until the epoxy set (about 10 minutes). After the hardening of the epoxy, the top surface of the Plexiglas dish was filled with 1 mL of saline. Using forceps under a microscope, the upper cuticle of the fly head was removed to form an opening. Air sacs and fat were then removed and muscles related to brain movement were cut. The brain-exposed fly fixed on the Plexiglas dish was mounted on a custom-made stand (Urin Seisakusho Co., Ltd.), and 1 mL of the saline was added on the Plexiglas dish. The saline consisted of following (in mM): 103 NaCl, 3 KCl, 5 TES [*N*-tris(hydroxymethyl)methyl-2-aminoethanesulfonic acid], 10 trehalose, 10 glucose, 7 sucrose, 26 NaHCO_3_, 1 NaH_2_PO_4_, 1.5 CaCl_2_, and 4 MgCl_2_ [80] adjusted to a pH of 7.25 with HCl and then filtrated. Osmolality of the saline was not adjusted (approximately 305 mOsm).

The GCaMP3-based fluorescent signals of the Or67d ORN-innervated glomerulus (DA1) in the antennal lobe [7, 19] (S3E and S3F Fig) were monitored using a microscope (BX51WI, Olympus) with a 40× water immersion objective (NA 0.8) (LUMPLFLN 40XW, Olympus), a mechanical shutter system (Physio-Tech), an ultraviolet (UV) laser unit, and a digital CCD camera (ORCA-R2, Hamamatsu Photonics). The signal was collected as monochromatic excitation light using imaging software (HCImage, Hamamatsu Photonics). Images were acquired at 512 × 512 pixels per 250 msec per frame. A single imaging cycle comprised three repetitions of 10 seconds (40 frames), signal collection with a 2-second air puff (frame 9–16), and a 20-second interval with the UV light shut off. Thus, 120 frames were obtained in the single imaging cycle. The laboratory made apparatus for air puffing consists of six components: an air tank with a regulator, a pump (PV820 Pneumatic PicoPump, World Precision Instruments), a flow meter (P-200-L0-4N-R2, Tokyo Keiso), an activated carbon filter, a vial with filter paper moistened by distilled water, and an air nozzle made from a hypodermic needle (NN-1838R, Terumo). All components were connected by silicon tubes. The air nozzle was placed 30 mm in front of the fly. Air was puffed at 30 mL/min (wind velocity: 0.18 m/sec).

Representative signal transitions were monitored during the continuous exposure for 15 minutes, and images were continuously acquired under UV irradiation. In all other cases, image acquisition and UV irradiation were not performed during the continuous exposure. Sequential switching between signal collection, UV irradiation, and air puffing was controlled by a stimulator (Master-8, A.M.P.I.). After imaging experiments in each fly, the maximum fluorescent response of DA1 glomeruli was measured for numerical correction of the neural response among individuals by exchanging 1 mL of the saline for an equivalent amount of 400 mM KCl solution on the fly fixed to the Plexiglas dish.

### Odor stimulation

cVA was diluted with hexane and stored in 1.5-mL tubes at -30°C. The cVA solution was stored on ice whenever used. Odor was delivered using a bench made cartridge consisting of toothpick tips and pipette (Fig 2A). One microliter of various dilutions of cVA was applied to the top of the toothpick. After evaporation of hexane, the cartridge covered with cVA was positioned between the air nozzle and antennae of the fly (Fig 2A) using a manipulator (M-3, Narishige) attached to a stand (Z-1, Narishige). cVA was delivered to the antennae with an air puff from the air nozzle. The cartridge was renewed after every single imaging cycle.

### Pharmacology

Picrotoxin (Sigma), CGP54626 hydrochloride (Tocris Bioscience), mecamylamine hydrochloride (Sigma-Aldrich), (+)-MK-801 hydrogen maleate (Sigma-Aldrich), and (+)-butaclamol hydrochloride (Sigma-Aldrich) were used as receptor antagonists of GABA_A_, GABA_B_, nicotinic acetylcholine, NMDA, and dopamine, respectively. Picrotoxin, mecamylamine hydrochloride, and (+)-MK-801 hydrogen maleate were dissolved in saline to 500 μM, 200 μM, and 200 μM, respectively. Both CGP54626 hydrochloride and (+)-butaclamol hydrochloride were dissolved in dimethyl sulfoxide (DMSO) to 100 mM and further diluted in saline to 100 μM. These drug solutions were stored at -30°C until use. During drug application, 1 mL of the saline applied on the fixed fly was first discarded and then equivalent amounts of drug solutions or saline as control were applied. Final concentrations of picrotoxin, CGP54626 hydrochloride, mecamylamine hydrochloride, (+)-MK-801 hydrogen maleate, and (+)-butaclamol hydrochloride were 250 μM, 50 μM, 100 μM, 100 μM, and 50 μM, respectively.

### Analysis and statistics

Fluorescent intensity on images of the DA1 glomerulus (S3E Fig) was converted into numerical values using a software (Image J 1.46r). F values were calculated by averaging values among the same frames in repetition of the single-imaging cycles. Δ*F/F* percentage values were then calculated using the formula

$$∆F/F\%=F_{t}/(\sum_{t=5}^{8}F_{t}/4)$$

where *Ft* is the averaged fluorescent intensity at a given time point (frame) of the single-imaging cycle.

Mean values in the peak fluorescent changes under each amount of cVA were used for fitting a logistic curve to data on the concentration-dependent response to cVA in Fig 2C. The curve was calculated with the formula

$$y=\frac{K}{1+be^{-cx}}$$

where *y* is the predicted value of the fluorescent change at a given amount of cVA (*x*), *e* is the Euler number, and *K*, *b*, and *c* are arbitrary constants. The optimal constants were calculated by the least squares method using the Solver in the Microsoft Excel for Mac 2011, which results in *K* = 47.919, *b* = 5.925, and *c* = 0.093.

Statistical analyses were performed with either JMP 8.0 (SAS Institute) or R version 3.0.2 [81]. Student's t-test with/without Holm's correction and Tukey honest significant difference (HSD) test with post-hoc test in ANOVA were applied to compare data between two groups and among multiple groups, respectively.

## Supporting Information

S1 Fig Male courtship latency in the presence of male-odor or cVA after habituation training. Courtship latency, which is the time lag to initiate courtship after pairing [82], was calculated from the observational data used in Fig 1C. The control mock male took more time to start courting in the presence of male odor or cVA, while the trained male showed undistinguished levels of latency regardless of the male-odor compared with no odor (no cVA) compared with solvent. Bars indicate mean ± SEM. Sol: solvent (hexane), cVA: 200 ng of cVA,-: no odor source, M: decapitated male. *, *P* < 0.05; ns, not significant from the Student's t-test.
(PDF)

S2 Fig Effects of 30 minutes of habituation training. Exposure to male-odor (left) or 200 ng cVA (right) for 30 minutes led to the effective habituation phenotype, in which high levels of courtship were elicited regardless of the presence of male-odor or cVA. Bars indicate mean ± SEM. Sol: solvent (hexane), cVA: 200 ng of cVA,-: no odor source, M: decapitated male, ns, not significant from the Student's t-test (*P* < 0.05).
(PDF)

S3 Fig Diagrams of the imaging analysis. A live fly was fixed on the imaging chamber based on the method by [83] for real-time imaging analysis. (A) The custom-made Plexiglas dish for fixation of the male fly. The dish (38 mm in diameter) has a dip (26 mm in diameter × 2 mm in depth) for application of saline and a hole (1.5 mm in diameter) for fixation of the fly. (B) Side view of a fixed male fly on the dish. (C) Ventral view of a fixed male fly on the dish. (D) Top view of the head of a fixed male fly under excitation irradiation. The brain was exposed by removal of the cuticle, air sac, and fat. cVA-responsive olfactory receptor neurons (Or67d ORNs) are visualized in the antennal lobe of a male fly using *Or67d-GAL4;UAS-GCaMP3*.*0*. The scale bar represents 200 μm. (E) Fluorescent image of the Or67d ORNs in the antennal lobe of a male fly. The framed area in (D) is magnified. The Or67d ORNs-innervated glomeruli (DA1) are indicated with dotted lines. Fluorescent signals in one of the glomeruli were monitored in the imaging analysis. The scale bar represents 50 μm.
(PDF)

S4 Fig Layout of the odor-stimulation system using a live male fly as an odor source. A live fly with wings and legs amputated was used as the odor source and fixed on the tip of the toothpick by epoxy.
(PDF)

S5 Fig Effects of neurotransmitter antagonists in neural desensitization of Or67d ORNs. (A) Schematic illustration of the application of antagonists to neurotransmitter receptors. Of the 2 mL of saline applied to the fly fixed onto the Plexiglas dish, 1 mL was first discarded and then an equivalent amount of drug solution or the saline as a control was applied and mixed well by gentle pipetting. (B) Application of saline as a procedure control decreased the odor responses of the Or67d ORNs (Dosed before CE). However, the desensitization effect by CE was clearly shown (After CE). (C-G) Effects of neurotransmitter antagonists were measured before dosing (Naïve), after dosing but before CE (Dosed before CE) and after CE. The input of GABA, an inhibitory neurotransmitter, was blocked by picrotoxin, an antagonist of the GABA_A_ receptor (C). CGP54626, a GABA_B_ receptor antagonist (D), dissolved in saline. Nicotinic acetylcholine receptor antagonists mecamylamine (E), NMDA (MK 801, F), and dopamine (butaclamol, G) were also used. Intensity of the response is presented as relative changes based on the response in naïve males. Significant differences are denoted by letters (Tukey HSD test; *P* < 0.05). Box plots represent 25th, 50th, and 75th percentiles with whiskers from minimum to maximum and mean with circles.
(PDF)

S6 Fig Courtship response to another male after habituation training. Courtship index (A) and latency (B) in male flies paired with another male. No increase of male-male homosexual courtship behavior was observed after 60 minutes of exposure to male odor, indicating that the odor habituation itself was not enough to mask all "maleness" of the target male. Bars indicate mean ± SEM. Male flies were housed in the upper cell of the chamber without (Mock) or with (Trained) another male fly in the lower cell for 60 minutes. ns: not significant from the Student's t-test (*P* < 0.05).
(PDF)
