# Supplementary figures and images for: Modification of Male Courtship Motivation by Olfactory Habituation via the GABAA Receptor in Drosophila melanogaster
Source: PLoS One. 2015 Aug 7;10(8):e0135186. doi: 10.1371/journal.pone.0135186 (PMC4529276; doi:10.1371/journal.pone.0135186)

S1 Fig.

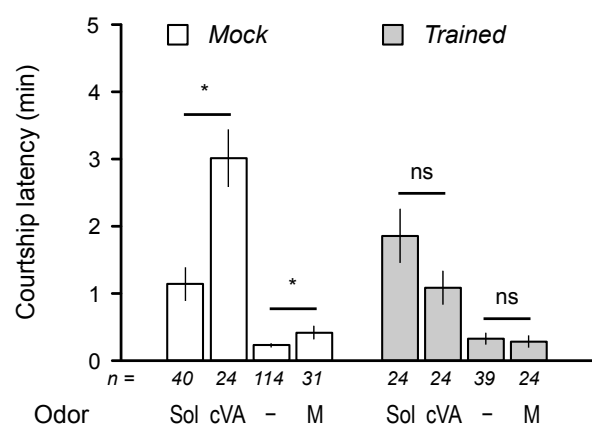

Supplement: S1 Fig — Courtship latency, which is the time lag to initiate courtship after pairing [82], was calculated from the observational data used in Fig 1C. The control mock male took more time to start courting in the presence of male odor or cVA, while the trained male showed undistinguished levels of latency regardless of the male-odor compared with no odor (no cVA) compared with solvent. Bars indicate mean ± SEM. Sol: solvent (hexane), cVA: 200 ng of cVA,-: no odor source, M: decapitated male. *, P < 0.05; ns, not significant from the Student's t-test. (PDF) [file pone.0135186.s001.pdf]

S2 Fig.

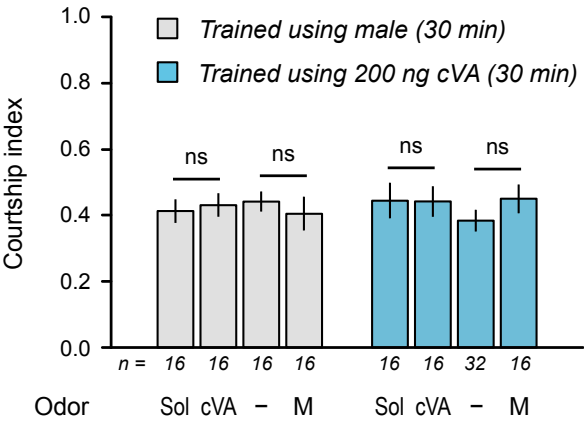

Supplement: S2 Fig — Exposure to male-odor (left) or 200 ng cVA (right) for 30 minutes led to the effective habituation phenotype, in which high levels of courtship were elicited regardless of the presence of male-odor or cVA. Bars indicate mean ± SEM. Sol: solvent (hexane), cVA: 200 ng of cVA,-: no odor source, M: decapitated male, ns, not significant from the Student's t-test (P < 0.05). (PDF) [file pone.0135186.s002.pdf]

S3 Fig.

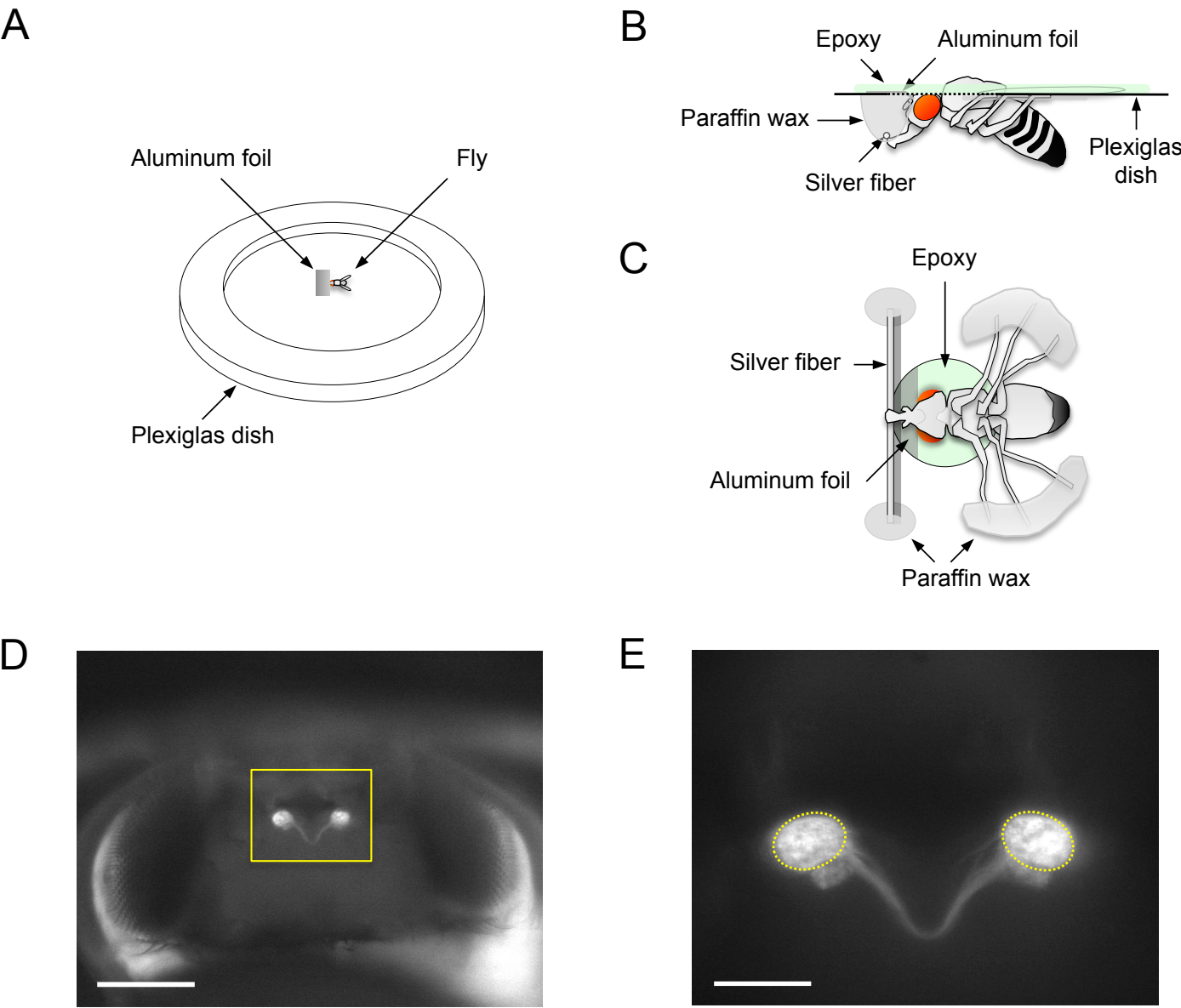

Supplement: S3 Fig — A live fly was fixed on the imaging chamber based on the method by [83] for real-time imaging analysis. (A) The custom-made Plexiglas dish for fixation of the male fly. The dish (38 mm in diameter) has a dip (26 mm in diameter × 2 mm in depth) for application of saline and a hole (1.5 mm in diameter) for fixation of the fly. (B) Side view of a fixed male fly on the dish. (C) Ventral view of a fixed male fly on the dish. (D) Top view of the head of a fixed male fly under excitation irradiation. The brain was exposed by removal of the cuticle, air sac, and fat. cVA-responsive olfactory receptor neurons (Or67d ORNs) are visualized in the antennal lobe of a male fly using Or67d-GAL4;UAS-GCaMP3.0. The scale bar represents 200 μm. (E) Fluorescent image of the Or67d ORNs in the antennal lobe of a male fly. The framed area in (D) is magnified. The Or67d ORNs-innervated glomeruli (DA1) are indicated with dotted lines. Fluorescent signals in one of the glomeruli were monitored in the imaging analysis. The scale bar represents 50 μm. (PDF) [file pone.0135186.s003.pdf]

S4 Fig.

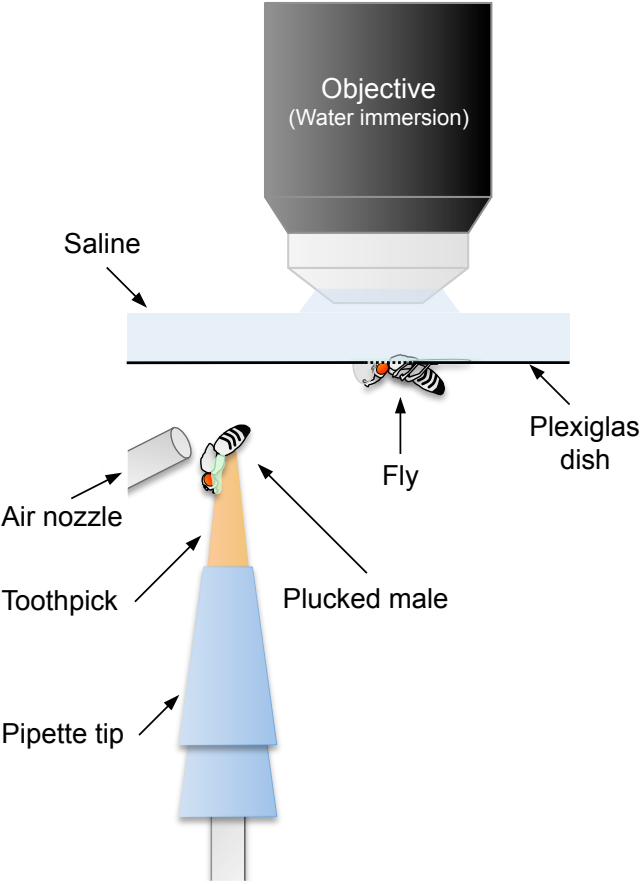

Supplement: S4 Fig — A live fly with wings and legs amputated was used as the odor source and fixed on the tip of the toothpick by epoxy. (PDF) [file pone.0135186.s004.pdf]

S5 Fig.

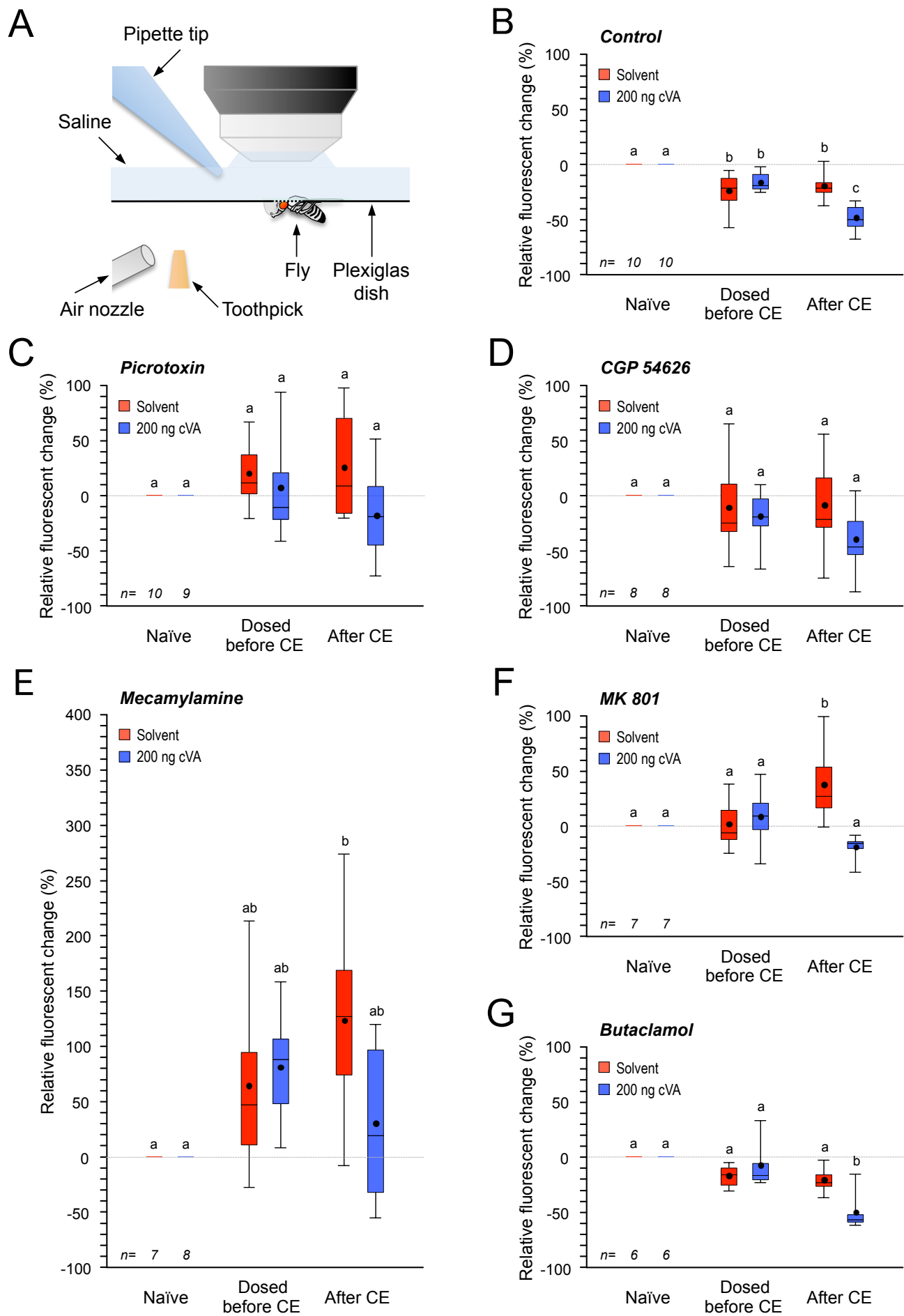

Supplement: S5 Fig — (A) Schematic illustration of the application of antagonists to neurotransmitter receptors. Of the 2 mL of saline applied to the fly fixed onto the Plexiglas dish, 1 mL was first discarded and then an equivalent amount of drug solution or the saline as a control was applied and mixed well by gentle pipetting. (B) Application of saline as a procedure control decreased the odor responses of the Or67d ORNs (Dosed before CE). However, the desensitization effect by CE was clearly shown (After CE). (C-G) Effects of neurotransmitter antagonists were measured before dosing (Naïve), after dosing but before CE (Dosed before CE) and after CE. The input of GABA, an inhibitory neurotransmitter, was blocked by picrotoxin, an antagonist of the GABAA receptor (C). CGP54626, a GABAB receptor antagonist (D), dissolved in saline. Nicotinic acetylcholine receptor antagonists mecamylamine (E), NMDA (MK 801, F), and dopamine (butaclamol, G) were also used. Intensity of the response is presented as relative changes based on the response in naïve males. Significant differences are denoted by letters (Tukey HSD test; P < 0.05). Box plots represent 25th, 50th, and 75th percentiles with whiskers from minimum to maximum and mean with circles. (PDF) [file pone.0135186.s005.pdf]

S6 Fig.

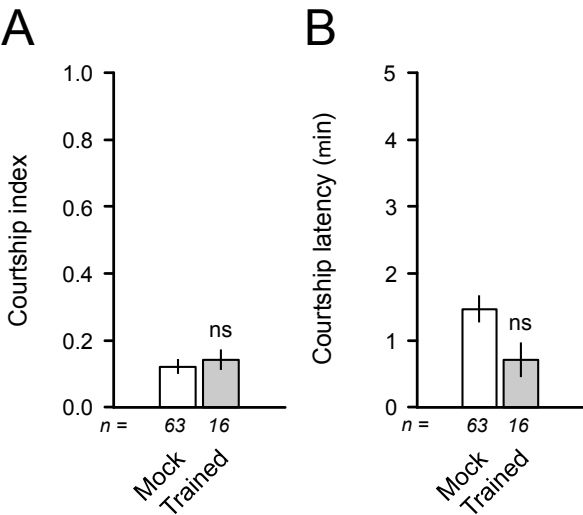

Supplement: S6 Fig — Courtship index (A) and latency (B) in male flies paired with another male. No increase of male-male homosexual courtship behavior was observed after 60 minutes of exposure to male odor, indicating that the odor habituation itself was not enough to mask all "maleness" of the target male. Bars indicate mean ± SEM. Male flies were housed in the upper cell of the chamber without (Mock) or with (Trained) another male fly in the lower cell for 60 minutes. ns: not significant from the Student's t-test (P < 0.05). (PDF) [file pone.0135186.s006.pdf]
